# Neuropathischer Pruritus – evidenzbasierte Behandlungsempfehlungen

**DOI:** 10.1007/s00115-022-01369-0

**Published:** 2022-08-11

**Authors:** Panoraia Baka, Frank Birklein

**Affiliations:** Klinik für Neurologie, Universitätsmedizin Mainz, Langenbeckstr. 1, 55131 Mainz, Deutschland

**Keywords:** Chronischer Pruritus, Neuropathischer Juckreiz, Gabapentinoide, Antidepressiva, Brachioradialer Pruritus, Chronic pruritus, Neuropathic itch, Gabapentinoids, Antidepressants, Brachioradial pruritus

## Abstract

Der neuropathische Pruritus ist ein bisher vernachlässigtes Symptom einer Vielzahl von neurologischen Erkrankungen. Mechanische Engpasssyndrome peripherer Nerven oder Nervenwurzeln, raumfordernde Läsionen des Zentralnervensystems, chronisch-entzündliche neurologische Erkrankungen oder eine Polyneuropathie können einen neuropathischen Pruritus verursachen. Selbst wenn die Identifizierung der zugrunde liegenden neurologischen Erkrankung erfolgreich ist, ist eine kausale Therapie nicht immer möglich, sodass eine effiziente symptomatische Behandlung die einzige therapeutische Option darstellt. Der Zweck dieser Übersichtsarbeit ist, die aktuelle Literatur zu verschiedenen Wirkstoffen und Therapieoptionen bei der Behandlung des neuropathischen Pruritus darzustellen.

## Abstract (en)

Neuropathic pruritus is a previously neglected symptom of a wide range of neurological diseases. Peripheral nerve or root compression syndromes, space-occupying lesions of the central nervous system, chronic inflammatory neurological diseases and polyneuropathy can cause neuropathic pruritus. Even when the identification of the underlying neurological disease is successful, a direct causal treatment is not always possible, hence an effective symptomatic treatment remains the only therapeutic option. The purpose of this review article is to present the current literature on various therapeutic agents and options in the treatment of neuropathic pruritus.

## Einleitung

Der neuropathische Pruritus ist ein bisher vernachlässigtes Symptom einer Vielzahl von neurologischen Erkrankungen [1]. Eine allgemein akzeptierte Definition gibt es bisher nicht, aber ähnlich wie die neuropathischen Schmerzen [2] entsteht der neuropathische Pruritus auch durch eine Läsion oder Erkrankung peripherer oder zentraler Strukturen des somatosensorischen Nervensystems. Beim Post-Zoster-Pruritus [3–5] und bei mechanischen Engpasssyndrome peripherer Nerven oder Nervenwurzeln, wie z.B. brachioradialem Pruritus [6–8], Notalgia paraesthetica [9, 10] sowie Pruritus bei Radikulopathien [11] ist der Juckreiz regional auf ein Innervationsgebiet begrenzt. Raumfordernde Läsionen des zentralen Nervensystems [12–20] und chronisch-entzündliche neurologische Erkrankungen [21, 22] sind seltene Ursachen eines neuropathischen Juckreizes mit variablem klinischem Erscheinungsbild. Immer häufiger wird ein generalisierter Pruritus einhergehend mit chronischen neuropathischen Schmerzen und Sensibilitätsstörungen als Symptom einer Polyneuropathie [23] und insbesondere der Small-fiber-Neuropathie [1, 24] erkannt. Im weiteren Sinne kann auch die Pathologie der intraepidermalen Nervenfasern bei dermatologischen Erkrankungen wie Keloiden [25] und Verbrennungen [4, 26, 27] unter dem Begriff neuropathischer Juckreiz eingeordnet werden.

Obwohl viele grundlegende Konzepte für die Entstehung des neuropathischen Juckreizes charakterisiert wurden, bleibt dessen Pathophysiologie unklar [28]. Eine häufige Überlappung von neuropathischem Pruritus und Schmerz [3, 29] hat das Forschungsinteresse geweckt und aufgrund dieser Gemeinsamkeit wurden Medikamente und Verfahren, die aus der Therapie neuropathischer Schmerzen bekannt sind, in der Behandlung des neuropathischen Juckreizes eingesetzt. In der aktuellen europäischen Leitlinie zur Behandlung des chronischen Pruritus findet sich nur eine Expertenempfehlung zur Behandlung des neuropathischen Pruritus mit Pregabalin oder Gabapentin [30]. Die Entwicklung einer wirksamen Strategie zur adäquaten Linderung von Juckreiz ist nicht nur für Dermatologen, sondern jetzt auch für Neurologen wichtig.

## Eingeschlossene Studien

Zur Identifizierung der einzuschließenden Studien wurden detaillierte Suchstrategien in den folgenden Datenbanken verfolgt: PubMed (National Library of Medicine, National Institutes of Health) und Scopus (Elsevier). Die Suchstrategien verwendeten eine Kombination von Schlüsselwörtern für die folgenden Begriffe: neuropathic pruritus/itch, neuropathic itch medication/treatment, notalgia paresthetica, post-herpetic pruritus/itch, post-burn pruritus, brachioradial pruritus. Diese Übersichtsarbeit umfasste 38 Veröffentlichungen, die insgesamt 459 erwachsene Patientinnen und Patienten und 41 Kinder mit neuropathischem Pruritus sowie 55 gesunde Probanden umfassten, bei denen experimentell induzierter Pruritus getestet wurde. Bis auf 2 doppelblinde, placebokontrollierte Studien [31, 32] handelt es sich bei den meisten Publikationen um Einzelfallberichte, Fallserien oder retrospektive Studien, die keine Metaanalyse erlauben.

### Gabapentinoide (Gabapentin, Pregabalin)

Gabapentinoide gehören zu den am häufigsten verwendeten Medikamenten zur Behandlung des chronischen Pruritus [33–37]. In einem Mausmodell mit durch Oxazolon induzierter chronischer atopischer Dermatitis war die Reduktion des Kratzverhaltens durch die Behandlung mit Pregabalin und Gabapentin auf die Bindung an die a2δ-1-Untereinheit der spannungsgesteuerten Kalziumkanäle im Spinalganglion zurückzuführen [38]. Die Wirkung von Pregabalin auf den Juckreiz nach Verbrennungen wurde in einer randomisierten, placebokontrollierten Doppelblindstudie untersucht. Neunzig Patienten, deren Körperoberfläche zu mehr als 5 % betroffen war, wurden eingeschlossen. Pregabalin wurde in einer Dosis von 75 mg 2‑mal täglich verabreicht und individuell auf 2‑mal 300 mg pro Tag erhöht. Hierbei wurde nach 28 Tagen eine signifikante Reduktion (*p* < 0,01) der Juckreizintensität bei Patienten in der Pregabalingruppe im Vergleich zur Placebogruppe beschrieben, insbesondere haben Patienten mit einer großflächigen Verbrennung davon profitiert [31]. Auch bei Kindern mit Verbrennungen scheint Gabapentin wirksam zu sein [39]. Dabei wurde berichtet, dass innerhalb von 24 h nach Beginn der Behandlung alle Kinder über eine deutliche Verringerung des Juckreizes berichteten, und so wurde die Einnahme von Antihistaminika reduziert. Ein gutes Ansprechen auf Gabapentinoide zeigten auch Patienten, mit einem seltenen hereditären Syndrom mit paroxysmalem neuropathischem Juckreiz, welches durch eine Variante im *SCN9A*-Gen (*SCN9A*: sodium voltage-gated channel alpha subunit 9; das für den Nav1.7-Natriumkanal codiert) verursacht wird [40], aber auch bei Patienten mit 2 seltenen Collagen Type VI Alpha 5 Chain gemäß COL6A5-Varianten mit chronischem proximal betontem Juckreiz und nachgewiesener Small-fiber-Neuropathie [41]. In einer weiteren, nichtrandomisierten Studie führte der Einsatz von Gabapentin zu einer signifikanten Verbesserung des Juckreizes bei 20 Patienten mit Notalgia paraesthetica, dabei lag der mittlere Juckreizwert vor der Behandlung bei 9,5 auf der visuellen Analogskala (VAS) und sank nachher auf 5 (*p* = 0,002; [42]). In einer Fallserie mit 14 Patienten mit multisegmentalem neuropathischem Pruritus aufgrund von degenerativen Bandscheibenveränderungen bemerkten 12 davon eine über 75 %ige Verbesserung nach der Behandlung mit Gabapentin (300–1200 mg täglich), davon 2 mit zusätzlicher Gabe von Mirtazapin (7,5 mg; [43]). Einzelne Fallbeobachtungen zeigen ebenfalls eine positive Wirkung von Gabapentin 300 mg 3‑ bis 6‑mal täglich auf den brachioradialen Pruritus [44].

### Antidepressiva

Für die orale Gabe von Antidepressiva gibt es nur Einzelberichte, z. B. zur Behandlung der Notalgia paraesthetica mit 10 mg Amitriptylin abends, hier zeigte sich eine Reduktion der Juckreizintensität von 7 auf 4 auf der VAS nach einer 3‑monatigen Behandlung [45]. Die lokale Anwendung von 1 % Amitriptylin/0,5 % Ketamin war im Einzelfall erfolgreich in der Behandlung des therapierefraktären brachioradialen Pruritus [46] und postherpetischen trigeminalen Pruritus [47]. Doxepin, ein trizyklisches Antidepressivum mit antihistaminischen Eigenschaften, wird lokal als 5 %ige Creme erfolgreich bei Patienten mit Juckreiz bei dermatologischen Erkrankungen wie der atopischen oder ekzematischen Dermatitis angewendet [48, 49]. Zur Behandlung des Pruritus nach Verbrennungen zeigte sich die lokale Anwendung von Doxepinsalbe in einer Studie wirksamer im Vergleich zu oralen Antihistaminika [50]. Hierbei berichteten 50 % der Patienten, die Doxepinsalbe verwendeten, einen kompletten Rücklauf des Juckreizes innerhalb von 3 Monaten im Vergleich zu nur 10 % der Patienten, die orale Antihistaminika einnahmen. Die verfügbaren Studienergebnisse sind jedoch widersprüchlich, da eine placebokontrollierte Studie [51] die Juckreizintensität nicht unterschiedlich (*p* = 0,994) zeigte, und eine Empfehlung kann hierzu nicht ausgesprochen werden.

### Capsaicin

Die lokale Applikation von Capsaicin, dem Inhaltsstoff scharfer Chilischoten, aktiviert die Transient Receptor Potential Vanniloid 1 (TRPV1)-Rezeptoren, die auf Keratinozyten und primär afferenten C‑Fasern vorkommen [32]. Der Behandlungserfolg nach einer 4‑wöchigen Behandlung mit 0,025 % Capsaicinsalbe bei Notalgia paraesthetica wurde bereits 1995 bei 70 % der mit Capsaicin behandelten Patienten vs. 30 % der mit dem Vehikel behandelten Patienten nachgewiesen [32]. Beobachtungsstudien und Fallserien sprechen für eine schnellere Wirksamkeit eines 8 %igen Capsaicin-Pflasters beim neuropathischen Pruritus [52–54] auch im Rahmen einer Small-fiber-Neuropathie [55]. Was noch offen bleibt, ist die Dauer der juckreizstillenden Wirkung, aufgrund pharmakologischer Überlegungen kann jedoch von einer Wirksamkeit von bis zu 3 Monaten ausgegangen werden.

### Lokalanästhetika

Lokalanästhetika wurden als Creme (2,5 % Lidocain und 2,5 % Prilocain [56, 57]), als 5 %iges lidocainhaltiges Pflaster [58] sowie regionalanästhetisch (Nervenblockade mit Tetracain und Bupivacain; [59, 60]) zur Behandlung des neuropathischen Pruritus versucht. In einer Studie mit 20 Patienten mit Notalgia paraesthetica wurde in 3 Sitzungen verdünntes 2 %iges Lidocain intradermal in den oberen Rückenbereich in Abständen von 1 cm um das betroffene Areal und segmental entlang der Dornfortsätze C2–T6 injiziert. Dadurch kam es zu einer signifikanten Reduktion der Juckintensität von 7 auf 1 auf der VAS nach der dritten Behandlung (*p* < 0,001; [61]).

### Botulinumtoxin A (BTX-A)

Botulinumtoxin blockiert die Freisetzung von Acetylcholin aus cholinergen Neuronen [62] und hemmt die Freisetzung von anderen Transmittern wie Glutamat, Substanz P und Calcitonin-Gen-verwandtem Peptid (CGRP) aus peripheren sudomotorischen und sensorischen Nervenendigungen [63, 64]. Experimentell konnte gezeigt werden, dass nach einem Histamin-Prick-Test die Behandlung mit BTX‑A (vs. Isotonische Kochsalzlösung) nicht nur die Juckintensität (*p* < 0,001), sondern auch die Juckfläche (*p* = 0,011), die Dauer des Juckreizes (*p* < 0,001), den Blutfluss und die Hauttemperatur verringerte (*p* < 0,001; [65]). Bei der Notalgia paraesthetica erbrachte eine Fallserie von jedoch nur 2 Patientinnen nach Injektion von 16 bis 25 U BTX‑A ein positives Ergebnis [66]. In einer kleinen, gut kontrollierten Studie mit 20 Patienten mit Notalgia paraesthetica hatte jedoch BTX‑A im Vergleich zu Placebo keinen Effekt gezeigt [67]. Wahrscheinlich effektiver ist BTX‑A bei Patienten mit Juckreiz nach Verbrennungen, wobei der juckreizstillende Effekt von BTX‑A ca. 9 Monate anhielt [68]. Interessant ist auch ein kürzlich veröffentlichter Fallbericht über die Linderung des schweren neuropathischen Juckreizes bei einem 58-jährigen Patienten mit primär progredienter multipler Sklerose durch lokale intradermale Injektionen von BTX‑A [22].

### Naltrexon

Morphin, pharmakologisch ein nichtselektiver Opioidrezeptoragonist, verursacht bei vielen Schmerzpatienten Juckreiz als Nebenwirkung [69]. Der genaue Wirkmechanismus ist noch nicht klar, aber in einer tierexperimentellen Studie mit intrathekal induziertem Morphin wurde gezeigt, dass es auf die µ‑Opioidrezeptoren auf spinalen inhibitorischen Interneuronen wirkt und zur Enthemmung des spinalen Juckreizkreislaufs führt [70]. Umgekehrt konnten Opioidrezeptorantagonisten das Kratzen auch bei experimentellem Juckreiz im Tierversuch unterdrücken [71]. In einer klinischen Studie mit 15 Patienten mit Pruritus nach Verbrennung gaben 72 % der Patienten an, dass sie mit der Linderung des Juckreizes durch Naltrexon zufrieden waren, und 69 % konnten die Einnahme anderer Medikamente zur Behandlung des Pruritus reduzieren oder absetzen [72].

### Transkutane elektrische Nervenstimulation (TENS) und kutane Feldstimulation (CFS)

Die TENS erwies sich in 2 Studien, eine davon randomisiert-kontrolliert, bei Patienten mit Juckreiz nach Verbrennungen als wirksam [73, 74]. Eine neue Methode, die kutane Feldstimulation (CFS), welche die C‑Fasern stimuliert [75], zeigte sich erfolgreich zur Linderung des Juckreizes bei brachioradialem Pruritus und Notalgia paraesthetica. Dabei ging die Reduktion des Juckreizes mit einer Degeneration epidermaler Nervenfasern einher, was durch den Verlust der Immunreaktivität des Protein-Genprodukts 9.5 in den Hautbiopsien zu Beginn und Ende der Behandlung belegt wurde [76].

### Akupunktur

In einer retrospektiven Analyse fand sich, dass Akupunktur bei 12 von 16 Patienten mit neuropathischem Juckreiz eine symptomatische Linderung des Juckreizes bewirkte [77]. Es gibt keine kontrollierten Studien zur Akupunktur bei neuropathischem Juckreiz. Bei atopischer Dermatitis war jedoch in einer kleinen randomisierten, kontrollierten Studie die Akupunktur mindestens so wirksam wie die Einnahme von Cetirizin [78].

## Weitere Therapiemöglichkeiten

In der Literatur finden sich noch folgende interessante Einzelberichte. Eine erfolgreiche Behandlung des Pruritus wurde bei einem Patienten mit amyotropher Lateralsklerose durch Cannabinoide [79] beschrieben. Eine Besserung des Juckens und des Kratzverhaltens ist bei einer Patientin mit postherpetischem Pruritus durch die intranasale Gabe von Butorphanol und Risperidon [80] dokumentiert. Butorphanol ist ein Opioid, das als Agonist auf die κ‑ und δ‑Opioidrezeptoren und als Antagonist auf die μ‑Opioidrezeptoren wirkt [81], und Risperidon, ein Neuroleptikum, wurde erfolgreich zur Behandlung von Patienten mit einer wahnhaften Parasitose eingesetzt [82, 83]. Bei einem Patienten mit Post-Zoster-Pruritus im Bereich des N. trigeminus führte die hochthorakale epidurale Infusion von Bupivacain und Clonidin zu einer Linderung der Symptomatik [84].

## Schlussfolgerung

Eine optimale Behandlung des neuropathischen Pruritus gibt es derzeit nicht. Noch fehlen größere, kontrollierte Medikamentenstudien sowie ein allgemein akzeptierter pathophysiologischer Ansatz. Wahrscheinlich ist ein multimodales Therapiekonzept ähnlich wie bei chronischen neuropathischen Schmerzen notwendig, jedoch auch dazu gibt es keine belastbaren Daten. Bis dahin sollten wir zumindest die vorhandene geringe Evidenz (s. Tab. 1), die in diesem Artikel dargestellt ist, nutzen, um erfolgreiche Behandlungskonzepte zum Wohle der Patienten zu erstellen.

**Table 1:** 

	Therapiemöglichkeiten	Quelle	Qualität der Evidenz
Postherpetischer Juckreiz	*2* *% Amitriptylin* *+* *0,5* *% ketaminhaltiges Gel* *5* *%iges Lidocainpflaster* über max. 12 h/d 4 *%iges Tetracain, gelöst mit 0,5* *%igem Bupivacain s.c.*: supraorbitale Injektion von 20 mg Tetracain in 0,5 ml 0,5 %igem Bupivacain	[47] [58] [59]	Fallbericht Fallbericht Fallbericht
Brachioradialer Pruritus	*1* *% Amitriptylin* *+* *0,5* *% Ketaminsalbe*: lokal 3‑ bis 4‑mal/d *8* *%iges Capsaicinpflaster* über ca. 60 min *Gabapentin* 900–1800 mg/d	[46] [52–54] [43]	Fallbericht Retrospektive Studie & Fallserien Fallserie
Notalgia paraesthetica	*Gabapentin* 300 mg/d für 4 Wochen *Amitriptylin* 10 mg/d für 9 Monate *0,025* *%ige Capsaicinsalbe* 3‑ bis 5‑mal/d für 4 Wochen *2* *%iges Lidocain intradermal* (1 cc) + 0,9 %iges NaCl (5 cc), 0,2–0,3 cc lokal in Abständen von 1 cm injiziert	[42] [45] [32] [61]	Nichtrandomisierte klinische Studie Fallbericht Doppelblinde RCT Fallbericht
Radikulopathien	*Gabapentin* 300–1200 mg/d	[43]	Fallserie
Z. n. Verbrennungen	*Pregabalin* 75 mg × 2, bis auf 300 mg/d *Naltrexon* 50 mg/d *BTX‑A* individuell *TENS* 1 h/d für 3 Wochen	[31] [72] [68] [73, 74]	Doppelblinde, placebokontrollierte Studie Fallserie Retrospektive Studie Pilotstudie, Fallbericht
